# Prevalence and Contributing Factors of Acne Vulgaris Among the General Population in the Jazan Region, Saudi Arabia: A Cross-Sectional Study

**DOI:** 10.7759/cureus.65407

**Published:** 2024-07-26

**Authors:** Amany Mashi, Sarah A Daghriri, Osama A Mobarki, Faisal Otaif, Osama A Suwaid, Rena H Alharbi, Khowlah A Adawi, Meshal A Alanazi, Mohammed Hurubi, Bayan A Qadiri, Almuhannad G Alnami, Bushra A Alfaifi, Ahmed Y Moafa, Haya A Alqahtani

**Affiliations:** 1 Department of Dermatology, Armed Forces Hospital, Jazan, SAU; 2 College of Medicine, Jazan University, Jazan, SAU; 3 College of Medicine, Jouf University, Sakaka, SAU

**Keywords:** saudi arabia, jazan region, treatment satisfaction, acne vulgaris, prevalence

## Abstract

Introduction: Acne vulgaris is one of the most prevalent inflammatory conditions in the world that primarily affects teenagers. Its prevalence and the contributing factors vary across different regions and populations. Genetic predisposition, hormonal influences, dietary habits, lifestyle choices, and environmental factors are believed to be significant contributors.

Methods: This was a cross-sectional study involving 419 participants from the Jazan region, Saudi Arabia. The study employed non-probability convenience sampling techniques. Data were collected through online questionnaires and analyzed using Statistical Product and Service Solutions (SPSS, version 27; IBM SPSS Statistics for Windows, Armonk, NY).

Results: The study found that acne prevalence was high, affecting more than half (66.8%) of the participants, with mild severity reported by 51.8%. Pimples were most commonly found on the face (65.2%), followed by the back (45.3%) and chest (29.6%). Participants with oily skin had twice the likelihood of acne compared to those with dry skin (OR=2.14). Increasing age was associated with a 5% decrease in acne risk per year. Significant associations were found for age (p=0.010), female gender (p=0.017), and oily skin (p<0.001) with acne development.

Conclusion: The study found a high prevalence rate of acne vulgaris among the young population in the Jazan region, Saudi Arabia. Age, female gender, and having oily skin were predictors for developing acne vulgaris. Complications such as acne scarring and psychological impacts such as shyness underscore the significant burden of acne on social and psychological well-being. Enhanced treatment and improved quality of life necessitate heightened awareness campaigns concerning acne vulgaris, its treatments, and associated complications, as revealed by the study.

## Introduction

Of the several cutaneous disorders that affect people, acne vulgaris is by far the most common [1]. Although it mainly affects people during their adolescence and young adult stages, it also affects a significant proportion of adults [2]. Acne vulgaris negatively affects patients’ quality of life because it significantly affects a person’s appearance. The desire to look good has been increasing in recent decades, with the advent of social media even putting more pressure on people to look good [3]. However, acne vulgaris is one of the leading conditions that negatively impact the quality of life, with studies indicating a prevalence rate of about 10% among the general population [3]. Among teenagers, the prevalence of acne vulgaris can range between 29% and 91% [4].

The predominant clinical manifestations of acne vulgaris encompass open and closed comedones, papules, pustules, nodules, cysts, and sporadic scarring [5]. Existing knowledge associates its causes with a complex pathophysiology that includes an unusually high production of sebum, anomalous keratinization, and inflammation of Cutibacterium acnes [6]. Risk factors of acne vulgaris range from genetic to hormonal and lifestyle factors, such as smoking and consuming a high-fat diet [7]. The relationship between acne and diet remains a highly debated topic, with some evidence indicating that, indeed, Western diet increases the possibility of having acne, especially among adolescents and young adults [8]. Other lifestyle choices have also been associated with acne, such as poor sleep and lack of physical exercise [8].

Traditionally, treatment for acne vulgaris has mainly been home-based. While home remedies still remain a popular treatment strategy, creams, and even surgical procedures have become increasingly common as the desire to look good increases [9]. However, the World Health Organization warns against possible drawbacks of certain treatment strategies, especially self-medication can lead to several issues, such as the misuse of topical and oral medications, which can lead to skin irritation and worsening of acne symptoms. Further, the suitable treatment may differ from one person to another based on various factors such as the causes, the patient's age, and the disease's severity. Therefore, part of treating acne vulgaris involves gaining an in-depth understanding of the condition and how it affects people of specific regions. The primary objective of this study is to ascertain the prevalence of acne vulgaris, identifying its contributing factors among the general populace of the Jazan region, Saudi Arabia.

## Materials and methods

Study design

This research employed a cross-sectional design and was conducted from November 2023 to April 2024 among the adult population of Jazan, Kingdom of Saudi Arabia (KSA). The study aimed to determine the prevalence of acne vulgaris and its contributing factors within the general population of the Jazan region in Saudi Arabia.

 Inclusion and exclusion criteria

The inclusion criteria for this study were individuals living in the Jazan region who agreed to participate and were 18 years of age or older. Conversely, the exclusion criteria encompassed individuals residing outside the Jazan region, those with severe psychiatric disorders, terminal illnesses, or specific chronic diseases. Additionally, individuals who do not speak or understand the Arab language were excluded to ensure accurate comprehension and response to the questionnaire.

Sampling technique

This study utilized a non-probability convenience sampling technique whereby respondents participated based on their availability and willingness to partake in the study during the designated period.

Sample size

Cochrane sample size formula was employed to ascertain the sample size. The formula is given as follows: n = Z2p (1 − p)/d2. Here, n represents the sample size, Z denotes the critical for 95% CI, 50% is the predetermined proportion, and d is the margin of error (5%) [10]. Initially, the minimum acceptable sample size computed was 384. Nevertheless, to bolster reliability, we chose to work with a slightly larger sample size of 419 respondents.

Data collection tools and procedures

The questionnaire was developed based on recent literature to align with the study's objectives. It was piloted with ten respondents to evaluate its readability and understandability. These pilot study participants were excluded from the main study. The reliability of the questionnaire was assessed using Cronbach's alpha, yielding a value of 0.845, showing a good level of internal consistency. Following the incorporation of adjustments prompted by their feedback, the questionnaire was disseminated among the adult populace residing in Jazan, Saudi Arabia, through WhatsApp, Facebook, and Telegram groups. The study included a section on the prevalence of acne and its potential complications. Acne severity was categorized into four levels: none, mild, moderate, and severe.

Data analysis

After completion of data collection, data were first entered into an Excel (Microsoft® Corp., Redmond, WA) database for the initial cleaning process, which included identifying and removing outliers, duplicates, and incomplete entries. Once cleaned, the data were coded and transferred to Statistical Product and Service Solutions (SPSS, version 27; IBM SPSS Statistics for Windows, Armonk, NY) software for analysis. Counts and frequencies were utilized to summarize categorical variables, whereas means and standard deviations were employed for continuous variables such as age. Multiple regression analysis was employed to investigate the relationship between various socio-demographic variables and the increased risk of acne vulgaris, and the odds ratio was used to determine the likelihood of developing acne associated with each variable with statistical significance determined at a threshold of p<0.05.

Ethical considerations

Ethical clearance was obtained from the Jazan ethics committee (reference no.: REC-45/11/1110) before data collection. The data collected adhered to anonymity by excluding personal identifiers, preventing any linkage between individual identities and study outcomes. The study respondents were informed about the study's purpose, procedures, associated risks, and potential benefits before consenting voluntarily, free from coercion.

## Results

Table 1 depicts the socio-demographic information of the participants. A total of 419 participants completed the online questionnaires. The mean age of the participants was 25.52±7.216. More than half 228 (54.4%) of the participants were males with the majority (284, 67.8%) of them being single, while 249 (59.4%) of them had a university education. Regarding employment, nearly half (206, 49.2%) of them were students with the vast majority (351, 83.8%) being non-smokers, 45 (10.7%) being smokers, and only 23 (5.5%) being previous smokers.

**Table 1:** Socio-demographic information of the participants (N=419) Socio-demographic information is presented in frequencies (n) and percentages (%).

Demographic information	Category	Frequency and Proportion, n (%)
Age (years)	Mean ± SD	25.52 ± 7.216
Gender	Male	228 (54.4%)
	Female	191 (45.6%)
Marital status	Single	284 (67.8%)
	Married	128 (30.5%)
	Divorced	7 (1.7%)
Education	Primary and secondary school	170 (40.6%)
	University	249 (59.4%)
Employment	Students	206 (49.2%)
	Employed	132 (31.5%)
	Unemployed	81 (19.3%)
Smoking	Non-smoker	351 (83.8%)
	Smoker	45 (10.7%)
	Previous smoker	23 (5.5%)

Table 2 depicts the acne prevalence and their sites among the participants. The results found a notable proportion (193, 46.1%) of the participants with oily skin type. More than half (280, 66.8%) of the participants had acne, while 139 (33.2%) of them had no acne. Of the participants, 217 (51.8%) had mild acne in terms of their severity. More than half (273, 65.2%) of the participants reported having pimples on their faces, nearly half 190 (45.3%) had them on their backs, and less than one-third (124, 29.6%) had a pimple on their chests.

**Table 2:** Acne prevalence and sites Participants’ acne prevalence and sites are presented in frequencies (n) and percentages (%).

Statements	Category	Frequency and Proportion n (%)
Skin type	Dry	36 (8.6%)
	Oily	193 (46.1%)
	Mixed	106 (25.3%)
	Normal	84 (20.0%)
Severity of acne	No acne	139 (33.2%)
	Mild	217 (51.8%)
	Moderate	55 (13.1%)
	Severe	8 (1.9%)
Is there any pimple in your face?	No	146 (34.8%)
	Yes	273 (65.2%)
Is there any pimple in your chest?	No	295 (70.4%)
	Yes	124 (29.6%)
Is there any pimple in your back?	No	229 (54.7%)
	Yes	190 (45.3%)

Table 3 depicts the participants’ acne complications. The findings revealed that the majority (211, 50.4%) of the participants had acne scarring, with a substantial proportion (197, 47.0%) of them reporting shyness and 183(43.7%) of them reporting post-inflammatory hyperpigmentation. Furthermore, nearly one-quarter (108, 25.8%) reported having depression, while only 76 (18.1%) of them reported social isolation.

**Table 3:** Acne complications Participants’ acne complications are presented in frequencies (n) and percentages (%).

Questions	Categories	Frequency and Proportion n (%)
Acne scarring	No	208 (49.6%)
	Yes	211 (50.4%)
Shyness	No	222 (53.0%)
	Yes	197 (47.0%)
Depression	No	311 (74.2%)
	Yes	108 (25.8%)
Social isolation	No	343 (81.9%)
	Yes	76 (18.1%)
Post-inflammatory hyperpigmentation	No	236 (56.3%)
	Yes	183 (43.7%)

Table 4 shows the distribution of acne complications among the age groups. Of the acne patients, 211 (50.4%) of them had acne scarring, while 197 (47.0%) of them had shyness. A total of 34 (17.3%) participants aged 20 years and below developed shyness compared to 94 (47.7%) aged 21-25 years and 69 (35.0%) aged above 26 years old. However, there was no significant difference in the development of acne scarring, depression, social isolation, and post-inflammatory hyperpigmentation across the three age groups.

**Table 4:** Acne complications among age groups Association between acne complications and the age groups. The chi-square test was used to determine statistical significance. P-value considered significant at p-value<0.05. *used to denote statistical significance.

Complications	Total, N (%)	Age groups			p-value
		20 years and below	21-25	Above 26 years	
Acne scarring	211 (50.4%)	50 (23.7%)	94 (44.5%)	67 (31.8%)	0.684
Shyness	197 (47.0%)	34 (17.3%)	94 (47.7%)	69 (35.0%)	0.003*
Depression	108 (25.8%)	22 (20.4%)	48 (44.4%)	38 (35.2%)	0.456
Social isolation	76 (18.1%)	12 (15.8%)	34 (44.7%)	30 (39.5%)	0.107
Post-inflammatory hyperpigmentation	183 (43.7%)	44 (24.0%)	88 (48.1%)	51 (27.9%)	0.091

Table 5 shows that age demonstrated a significant association (OR: 0.95, 95% CI: 0.90-1.00, p=0.010), indicating that, for each additional year of age, the odds of having acne decrease by 5%. Gender also revealed a significant difference, with females exhibiting a 2.09 times higher likelihood of acne compared to males (95% CI: 0.499-1.128, p=0.017). The skin showed significant associations, where individuals with oily skin had 2.34 times higher odds of acne (95% CI: 1.15-4.32, p<0.001) compared to those with dry skin. Similarly, the presence of pimples on the face (OR: 34.488, 95% CI: 21.616-68.528, p<0.001), chest (OR: 3.789, 95% CI: 2.206-6.510, p<0.001), and back (OR: 4.100, 95% CI: 2.597-6.472, p<0.001) were strongly associated with increased odds of acne. The other demographic variables did not show any statistically significant differences with acne.

**Table 5:** Logistic regression analysis for factors associated with acne Association between socio-demographic variables and the increased risk for acne vulgaris. Logistic regression was used to determine significance. P-value considered statistically significant at p<0.05. *used to denote statistical significance.

Factors		OR	95% CI for OR		P-value
			Lower	Upper	
Age		0.95	0.90	1.00	0.010*
Gender	Male	1.00			0.017*
	Female	2.09	0.499	1.128	
Marital status	Single	1.00			
	Married	2.11	0.53	2.76	0.875
	Divorced	1.76	0.43	2.69	0.653
Education	Primary and secondary	1.00			0.069
	University	1.464	0.970	2.210	
Employment	Student	1.00			
	Employed	1.14	1.08	4.43	1.35
	Unemployed	1.72	1.19	2.76	1.40
Smoking	Non-smoker	1.00			
	Smoker	1.37	0.67	2.73	0.621
	Previous smoker	3.26	0.81	5.41	0.617
Skin type	Dry	1.00			
	Oily	2.34	1.15	4.32	<0.001*
	Mixed	1.62	0.68	2.69	0.432
	Normal	2.11	0.97	2.93	0.323
Pimple on the face	No	1.00			<0.001*
	Yes	34.488	21.616	68.528	
Pimple on the chest	No	1.00			
	Yes	3.789	2.206	6.510	<0.001*
Pimple on the back	No	1.00			
	Yes	4.100	2.597	6.472	<0.001*

## Discussion

The study reported 280 (66.8%) prevalence of acne among the participants of the Jazan region, KSA. The prevalence rate observed in this study slightly exceeds the 65% acne prevalence from a study by Alshammrie et al. in Hail, Saudi Arabia [11]. Conversely, Alajlan et al.'s study in the KSA reported a lower prevalence of 55.5%, contrasting with the findings of the present study [12]. The differences in the prevalence rate across the three studies could be explained by the differences in the sample sizes and the age distribution of the participants considering that acne is common to people aged up to 30 years [13].

The majority of the participants reported having pimples mainly on their faces (273, 65.2%), followed by the backs (190, 45.3%) and the chest (124, 29.6%). The findings are consistent with those of a study conducted in the KSA by Allayali et al., which reported the face to be the most common part of the body affected by acne, followed by the back and the chest [14]. The study noted that slightly more than half (217, 51.8%) of the participants had mild acne in terms of their severity. The findings are in line with those of a study conducted by Di Landro et al. who reported the existence of mild-to-moderate acne in majority of the adolescents and young adults [15]. Regarding complications, the majority (211, 50.4%) of the participants reported acne scarring with a notable proportion of 197 (47.0%) reporting shyness. The study noted that 34 (17.3%) of the participants aged 20 years and below developed shyness compared to 94 (47.7%) aged 21-25 years and 69 (35.0%) aged above 26 years old. Similarly, a study conducted by Tan et al. noted that the majority of participants expressed social stigma associated with their skin condition which brought about shyness [16].

Our results revealed that an increase in age by one year reduced the chances of developing acne by 5%. Furthermore, it was revealed that being a female was associated with two times increased risk of developing acne; consistent with the findings by Bagatin et al. who found females to be at a higher risk of developing acne than males due to fluctuations in hormone levels during menstrual cycles [17]. Having oily skin, pimples on the face, pimples on the chest, and pimples on the back were statistically associated with acne. The findings concur with those of the study conducted by Alshammrie et al. which reported a statistically significant association between oily skin and pimples on the face with the development of acne [11]. With a substantial proportion of 197 (47.0%) of the participants reporting feeling shy because of acne, the condition can affect social life, and self-esteem and can even result in long-term psychological effects. This explains the need for proper management plans for treatment of the condition and its complications.

One of the investigation’s limitations was the use of a descriptive study approach, which can only identify relationships between attributes, not causalities. Considering that the study was conducted using online surveys, bias may have resulted from respondents' correct recording of their answers without a means of verifying them. This was mitigated by piloting the questionnaire with a small group to refine question clarity. Additionally, because the study was limited to the Jazan region, its conclusions cannot be applied to the whole population of Saudi Arabia. Longitudinal studies could span over time, allowing for the exploration of causal relationships between variables.

## Conclusions

In conclusion, this study highlights a significantly high prevalence of acne vulgaris among the population in the Jazan region of Saudi Arabia, with distinct risk factors such as age, female gender, and oily skin contributing to its development. Mild acne severity was prevalent among more than half of the participants. Complications such as acne scarring and psychological impacts such as shyness underscore the significant burden of acne on social and psychological well-being. Acne scarring and associated complications such as shyness were common, particularly among younger age groups. The study finds the need for increased awareness efforts through targeted educational campaigns that focus on acne prevention, treatment options, and the importance of early intervention.
